# Translational research in sepsis - an ultimate challenge?

**DOI:** 10.1186/2040-7378-3-14

**Published:** 2011-11-14

**Authors:** Tim G Kampmeier, Christian Ertmer, Sebastian Rehberg

**Affiliations:** 1 Department of Anesthesiology and Intensive Care, University Hospital of Muenster, Muenster, Germany

**Keywords:** randomized controlled trials, sepsis, septic shock, translational research

## Abstract

In the era of evidence-based medicine, large, randomized, controlled, multicenter studies represent the "summit of evidence". In contrast to specialties like cardiology, the majority of randomized, controlled trials in critical care medicine, however, have failed to demonstrate a survival benefit; notably, despite encouraging results from experimental and phase-II clinical studies. The difficulty in translating our theoretical knowledge into successful multicenter randomized, controlled trials and subsequent treatment recommendations may represent one reason, why the mortality of septic shock still averages between 40-60%, although our knowledge about the underlying pathophysiology has considerably increased and international guidelines have widely been implemented. The present article elucidates some of the difficulties in translating research from bench to bedside.

## Introduction

In the era of evidence-based medicine, large, randomized, controlled, multicenter studies (together with meta-analyses) represent the "summit of evidence" [1]. In contrast to specialties like cardiology, the majority of randomized, controlled trials (RCT) in critical care medicine, however, have failed to demonstrate a survival benefit [2]; notably, despite encouraging results from experimental and phase-II clinical studies. The difficulty in translating our theoretical knowledge into successful multicenter RCTs and subsequent treatment recommendations may represent one reason, why the mortality of septic shock still averages between 40-60% [3], although the understanding of the underlying pathophysiology has considerably increased and international guidelines have widely been implemented.

Just two examples for this dilemma: In 1995, Hebertson et al. described an attenuation of the decrease in left-ventricular contractility by tumor necrosis factor-alpha-(TNFα) antibodies in endotoxemic pigs [4]. In the same year, Givner and colleagues reported a reduction in mortality of newborn rats with group B streptococcal disease due to the use of TNFα-antibodies [5]. The subsequent pilot study on nine patients with septic shock revealed no side effects concerning the use of TNFα-antibodies as an adjunct therapy regardless of the administered dose [6]. Only a few months later, the RCT with 141 patients receiving either placebo or TNF-receptor:Fc fusion protein in three different dosing regimens not only failed to show any survival benefit, but even suggested an increase in mortality associated with higher doses [7].

The "VAsopressin and Septic Shock Trial" (VASST) represents another, more recent example. Whereas there was consistent and extensive evidence from numerous experimental [8,9] as well as small clinical trials [10-12] about the efficacy and benefit of a supplementary low-dose infusion of arginine vasopressin (AVP) on catecholamine requirements and several other outcome measures in septic shock, the large, multicenter VASST study revealed no significant difference in mortality between sole norepinephrine and combined AVP and norepinephrine in the overall population [13].

But even if the initial RCT has been successful, like for the use of stress-dose corticosteroids in septic shock [14], confirmation studies may turn out negative [15]. This may lead to a so-called "pendulum effect" [16], thereby leaving clinicians in frustration and uncertainty about how to treat their patients. The present article elucidates some of the difficulties in translating our knowledge from bench to bedside.

## Basal differences between experimental and clinical research

First of all, we should keep in mind the elementary differences between experimental and clinical trials: preclinical studies are usually performed in young and healthy animals, whereas the majority of patients in the intensive care unit (ICU) suffer from multiple and severe co-morbidities such as chronic heart failure, diabetes, chronic kidney failure. Furthermore, preclinical research allows a well-defined injury (e.g. endotoxemia, cecal ligation and puncture, pneumonia) and perfectly standardized therapeutic interventions. Contrary, study populations of large RCTs are characterized by different sources of sepsis (e.g. pneumonia, abdominal infections, surgical site infections) and concomitant therapies are mostly left to the discretion of the attending physician. To increase the clinical relevance of experimental research, it has been suggested to use "higher fidelity animal models" in the future [17]. Among others, these are characterized by inclusion of older animals and different genetic lines. The fidelity is further increased by the use of "two-hit models" (e.g. pneumonia after following a trauma or burn injury) that mimic nosocomial sepsis secondary to an initial insult more realistically than "one-hit models".

An elementary difference between research in cardiology and critical care is that the pathophysiology and clinical symptoms between different patients with myocardial infarction, for example, are almost identical and quite specific, whereas critical care syndromes like the "systemic inflammatory response syndrome" (SIRS) or "acute respiratory distress syndrome" (ARDS) can be caused by numerous pathophysiological pathways and their diagnosis is based on rather unspecific definitions. As a consequence, the development of definite treatment strategies is more difficult. This hypothesis is supported by the fact that the current guidelines of the Surviving Sepsis Campaign contain 53 class I recommendations with only 8 being classified as grade A, whereas the guidelines for the treatment of ST-segment elevation myocardial infarction, for example, include 93 class I recommendations. Against this background, the first step in improving translational research in critical care is to increase the knowledge of the underlying pathophysiology by experimental research.

## Patient characteristics in RCTs

A major problem of clinical RCTs is represented by their heterogeneity in several aspects: the severity of illness, the source of infection, the timing of intervention, and concomitant therapies. Severe sepsis and septic shock are associated with a different baseline risk of death. Inclusion of both, severe sepsis and septic shock, without clear differentiation will mix up the effects on both patient cohorts. In addition, from a statistical point of view demonstrating a survival benefit becomes more difficult, if the mortality in the control group is reduced by including patients with septic shock and severe sepsis as compared to a control group including only septic shock patients. Furthermore, RCTs often not only include patients with sepsis deriving from different foci (e.g. pneumonia, intraabdominal sepsis, surgical site infection) but also patients with systemic inflammation due to a non-septic cause (e.g. pancreatitis, post-cardiopulmonary resuscitation, burns). These different pathophysiological pathways further increase the heterogeneity of the study population and, thereby, the risk of failure.

Since it may be assumed from the past that specific interventions are likely to fail if tested in a heterogeneous population (so-called "one-size-fits-all concept"), it appears appropriate to target study interventions for selected patient cohorts (e.g. patients with postoperative, ventilator-associated pneumonia). In these highly selected cohorts, it is more likely to unmask a specific intervention as "clearly beneficial", "clearly harmful", or "probably futile". Such a concept seems to be successful in other specialties with generally stricter inclusion criteria [18]. On the downside, trials with very strict inclusion criteria are likely to be terminated due to slow recruitment. It is one major goal for the next years to find the right balance in defining severity of illness and patient characteristics for successful RCTs [19].

## Timing of the study intervention

Closely associated with the severity of illness is the timing of intervention, i.e. the time window from the point of diagnosis up to study inclusion and initiation of the therapy. On the one hand, a wide time window for inclusion, may increase the chance to recruit more patients in a shorter time period. On the other hand, more patients will present with progressive disease and even multiple organ failure at the start of treatment, which may negatively influence the potential benefit of any intervention [19].

A recent negative trial of fluid therapy in severe sepsis recruited patients who already fulfilled the predefined criteria of normovolemia prior the inclusion [20], thereby representing an example of suboptimal patient selection. If e.g. two fluids are compared for volume therapy, it is essential to include patients in the very early phase of disease, in which they are actually hypovolemic. In normovolemic patients, however, any excessive fluid therapy regardless of the individual type may only proof deleterious [21].

Several clinical trials of anti-inflammatory mediators used liberal inclusion windows [22-24] notwithstanding the fact that the extent of inflammation in critically ill patients depends on the time elapsed since the initial injury [25]. The typical time course starts with an early phase of hyperinflammation, where anti-inflammatory mediators may theoretically be promising [26], and a prolonged period of immunosuppression, where anti-inflammatory treatment may supposedly be detrimental.

Acquisition of patients in the very early phase of critical illness requires optimal research infrastructure with minimal administrative burden. Since the most severely ill patients need immediate treatment at the time of admission, a delay of several hours until the start of study intervention may miss its "golden hour" and may be inadequately judged as futile or harmful.

## Heterogeneity in concomitant treatments and end point selection

The heterogeneity in concomitant treatments represents another very important confounder. Whereas most sepsis trials provide more or less strict guidelines for the examined intervention, the concomitant treatment is mostly left to the discretion of the attending physician. It is obvious, that controversially discussed and differently handled measures like the type of fluid or vasopressor agent used, the antibiotic regimen, hand hygiene compliance, prevalence of multidrug-resistant microbials, ventilatory settings, sedation strategies, glucose control, ulcer or thromboembolic prophylaxis may influence the patients' outcome in a significant way. These therapeutic strategies often differ not only between countries, but also between hospitals and even doctors [1,19]. Notably, these confounding differences in co-treatments may have a considerable higher impact on the trial endpoint than the study intervention itself.

Therefore, relevant concomitant treatment strategies should be defined as strictly as the investigated therapy. The guidelines of the Surviving Sepsis Campaign represent an international standard for some but not all co-treatments [27]. At least, there should be a minimum standard guaranteed in all institutions, i.e. by applying simple checklists like the "fast hug" system [28]. Another way of controlling heterogeneous co-interventions is to investigate not a single but a bundle of therapeutic interventions, e.g. the "sepsis bundles" [29], or defined protocols, such as the "early goal directed therapy" approach by Rivers et al. [30].

Finally, the problem of selecting appropriate end points for RCTs should be addressed. Mortality still represents the most impressive and the most objective one. However, even this "unassailable" end point is associated with some limitations. As discussed earlier, potential beneficial effects of a treatment can be neutralized, if patient populations are not defined adequately. In addition, death in the ICU is often preceded by end-of-life decisions. This process may not influence the absolute rate of mortality but potentially the time of death and thereby survival time. Furthermore, a reduction in mortality does not provide any information about the mechanism of action of the interventions studied. Therefore, alternative or secondary outcome variables are warranted. Organ function scores, ventilator-free days, ICU stay, health-adjusted quality of life or quality-adjusted life years represent some examples. However, the value of differences in these secondary outcomes without changes in mortality remains to be determined.

## Future perspectives

Last but not least the increasing relevance of theragnostics should be mentioned. Theragnostic is best described as the use of biomarkers to identify patients, who are most likely to benefit from a certain intervention. In septic patients, procalcitonin represents an example for a biomarker that can be used to guide antibiotic treatment [31]. This approach of individualized medicine can be extended to pharmacogenomic biomarkers that give information about the probability of success for the individual compound, similar to the treatment of cancer.

In this context, genetic differences between individuals will increasingly influence and potentially guide therapies in critical care. Concerning the use of AVP in septic shock patients, Dr. Nakada and colleagues reported that a specific genetic variation in leucyl/cystinyl aminopeptidase (=vasopressinase, the enzyme that metabolizes AVP) is associated with 28-day mortality in septic shock and with biologic effects on AVP clearance [32]. By determining this genetic variation, the probability of success of AVP therapy could be specified. In addition, dose selection of AVP might be guided by this knowledge. For example, if a patient has a genetically determined, increased AVP clearance, higher doses might be chosen for the treatment than for a patient with a low AVP clearance.

## Conclusions

In summary, improvement of translational research in sepsis and critical illness should consist of a bi-lateral approach. On the one hand, the clinical relevance of preclinical studies can be increased by the use of "high-fidelity" and "two-hit" animal models. On the other hand, RCTs should be designed to optimize time of study intervention, limit heterogeneity in patient characterization, standardize concomitant treatments and investigate not a single but bundles of interventions. As a consequence, RCTs will probably become smaller in sample size, but hopefully will provide more valuable evidence for the benefit of our patients.

## Competing interests

The authors declare that they have no competing interests.

## Authors' contributions

TGK, CE and SR wrote, edited and approved the manuscript.
